# Ochratoxin A Contamination of Food from Croatia

**DOI:** 10.3390/toxins2082098

**Published:** 2010-08-10

**Authors:** Maja Peraica, Dubravka Flajs, Ana-Marija Domijan, Dario Ivić, Bogdan Cvjetković

**Affiliations:** 1 Unit of Toxicology, Institute for Medical Research and Occupational Health, Ksaverska c. 2, 10000 Zagreb, Croatia; Email: dflajs@imi.hr (D.F.); adomijan@imi.hr (A.-M.D.); 2 Department of Plant Pathology, Faculty of Agriculture, Svetošimunska 25, 10000 Zagreb, Croatia; Email: divic@agr.hr (D.I.); bogdan.cvjetkovic@zg.t-com.hr (B.C.)

**Keywords:** beans, endemic nephropathy, maize, ochratoxin A, wheat, wine

## Abstract

Ochratoxin A (OTA) is a mycotoxin with nephrotoxic, genotoxic and carcinogenic properties produced by *Penicillium* and *Aspergillus* moulds under different climatic conditions. Humans and animals are exposed to this compound mainly via ingestion of contaminated food. In Croatia, research on mycotoxins focused on OTA when the mycotoxin theory of endemic nephropathy (EN) was postulated. Ochratoxin A was more frequent and at higher concentration in foods from EN than those from the control regions. Subsequently, OTA concentrations were determined in some commodities intended for human consumption such as maize, wheat, beans and wine. Samples from all parts of Croatia were analyzed and OTA was found in all types of commodities. It was frequently found together with other mycotoxins (fumonisin B_1_, fumonisin B_2_ and zearalenone). In general, OTA concentration in foods from Croatia is low, but the frequency of positive samples shows considerable variations from year to year depending also on sampling location. Although low levels of OTA were found in a large proportion of analyzed food samples, its persistent co-occurrence with other significant mycotoxins should raise serious public health concerns as there interactions may be synergistic or additive in causing toxicity in humans and animals. There is need to establish control measures through which such contaminations in foods can be managed.

## 1. Introduction

Ochratoxin A (OTA) is a mycotoxin produced by *Penicillium* and *Aspergillus* moulds. It contaminates cereals, cereal products, coffee, nuts, meat, milk, and other commodities like wine, beer and cheese under different climatic conditions [1]. Although there are some reports of possible air-borne OTA intoxication, humans and animals are exposed to this mycotoxin mainly via ingestion of foods contaminated with OTA [2]. In some European countries it is estimated that commodities that contribute to OTA exposure are contaminated cereal (58%), wine (15%) and pork meat (3%)[3,4,5].

In Croatia, as well as in some countries in the tributaries of the Danube, a human kidney disease known as endemic nephropathy (EN), still of unknown origin, is prevalent. In the case of Croatia, EN occurs in 14 villages in the geographically limited area of Brodsko-posavska County, with a population of about 10,000 inhabitants. In this area the occurrence of upper urothelial tumors among humans is about 55 times higher than in other parts of Croatia, while the incidence of urinary bladder tumors is similar to the rest of the country [6]. The increased frequency of tumors where EN is prevalent indicates that some environmental factors are involved in the development of both diseases [7]. One of the theories yet still plausible, is that OTA (alone or together with other mycotoxins) may be involved in the development of these diseases. This theory was initially postulated based on the histological similarities observed in the kidneys of pigs with porcine nephropathy fed OTA contaminated feeds in Scandinavian countries as well as those seen in the kidneys of humans with EN [8]. Both nephrotoxic and carcinogenic properties of OTA have been demonstrated in a number of experimental and domestic animals [3,9]. In Croatia, both field and experimentation studies on OTA were conducted with the aim to establish whether exposure to OTA is the major cause of BEN and related tumors to resolve this public health problem. 

In fact, OTA was first found in human blood when samples from Croatian EN region were analyzed [10]. Subsequently, it was found that OTA is more prevalent and at higher concentrations in human blood samples from EN than those from control regions [11,12]. This corroborated the mycotoxin theory of the etiology of EN. However, low concentrations of OTA were frequently found in blood of humans in countries where the EN is not present [13] but it is not clear whether this has any toxicological significance. The genotoxicity of OTA was demonstrated in experimental animals even treated orally with 5 ng/kg body weight, which is estimated human daily intake typical for a European diet [14,15,16]. 

*Aspergillus* and *Penicillium* moulds may, under certain environmental conditions, simultaneously produce mycotoxins other than OTA. On the other hand, it is not common to find food contaminated with only a single mould, and it is equally possible that these fungi produce multiple mycotoxins, which may cause both additive and synergistic effects in animal and man [14,17,18]. Thus, OTA usually contaminates food commodities together with other mycotoxins such as citrinin, fumonisin B1, fumonisin B2 and zearalenone.

Commodities such as bread, beans and wine are commonly consumed in large amounts in Croatia and presence of OTA in these commodities may result in considerable increased human exposure to this mycotoxin. In as much as there are limited data available in certain circumstances, this review is aimed to critically evaluate the occurrence of OTA in different commodities for human consumption from both EN and non-EN regions of Croatia. Other mycotoxins, analysed together with OTA in studies reported in this review will also be mentioned in cases when data is available. This review is intended to show that data on the co-contamination with OTA and other mycotoxins are quite rare albeit the possibility of synergistic or additive toxic effects seen on experimental animals. Data on OTA contamination of various commodities from Croatia discussed in this review are summarized in Table 1.

## 2. OTA Food Contamination

### 2.1. Wheat

There are few reports on wheat contamination by OTA. The first report on wheat from EN region of Croatia contaminated with OTA was presented by Pavlović *et al.* [19]. In that work 130 wheat field samples were collected in the period 1972–1976 and analyzed via thin layer chromatography (TLC) at a detection limit of 5 µg/kg. As found overall, 8.5% of samples had OTA and the percentage of positive samples in this period ranged from 0 (1973) to 25% (1972). The quantification of OTA performed in six positive samples revealed that the highest OTA concentration was 140 µg/kg, and one third of these samples contained levels above 100 µg/kg. In a more recent study, concentrations of OTA in wheat samples from both EN and non-EN of Croatia were determined [20]. Accordingly, samples collected from Brodsko-posavska county, a region with prevalence of endemic nephropathy (EN region), showed significantly (P < 0.001) higher mean OTA concentration of 38.8 ± 27.1 µg/kg (range: 0.02–160.0 µg/kg) than samples from non-EN regions of Croatia. Further data from this study [20] revealed mean OTA concentrations in wheat samples from Osijek had significantly (P < 0.01) higher contamination levels of OTA of 8.7 ± 8.2 µg/kg when compared with those of Hrvatsko Zagorje and Istria that had mean levels of 2.1 ± 1.5 µg/kg and 1.3 ± 2.6 µg/kg, respectively. Such data indicate that OTA contamination of wheat as seen varies from one region to the other possibly due to climatic conditions. 

### 2.2. Maize

Like wheat, maize contamination with OTA was the first time reported in Croatia by Pavlović and coworkers [19]. In view of the fact that Pavlović *et al.* [19] analyzed 542 maize samples from EN regions for OTA between 1972 and 1976 and using TLC found that 45 (8.3%) of these samples contained OTA above detection limit (5 µg/kg) with two of positive samples having OTA levels above 100 µg/kg. The frequency of positive samples varied significantly from year to year ranging between 0 (1976) to 15% (1972). In the related study, OTA contamination of maize from EN and non-EN regions of Croatia between 1996 and 1997 was measured [21]. Accordingly, in total, 209 samples were collected over a year to year period from these regions and analyzed for OTA using high performance liquid chromatography (HPLC) coupled with fluorescence detection (detection limit of 0.2 ng/g). OTA concentration was not significantly different in samples collected in endemic and non-endemic regions in 1996. Whereas in 1997, a significantly (P < 0.01) much higher mean content (73.4 µg/kg) of OTA was observed in 50% of maize samples from EN region and much lower OTA mean level of 20.2 µg/kg was found in 20.3% of maize samples from non-EN region of Croatia. Further data also revealed that co-contamination of OTA with FB_1_ and FB_2_ was observed in 95% of maize samples analyzed over the two year period [21]. Although no regional variation was observed, mean FB_1_ + FB_2 _content (134 µg/kg) in maize samples collected during 1997 was significantly lower than those of 1996 (645 µg/kg). This study was the first to demonstrate that maize from Croatia may be contaminated not only with one, but several mycotoxins.

In another study, maize from the county of Slavonski Brod, an area with endemic nephropathy (EN region) and non-EN region of Croatia was collected [20]. Data obtained in this study indicated that maize samples from EN region of Slavonski Brod had significantly (P < 0.001) much higher mean OTA concentration of 20.0 ± 14.8 µg/kg than that of maize samples from Osijek (0.8 ± 1.3 µg/kg), Hrvatsko Zagorje (0.4 ± 0.4 µg/kg) or Istria (0.4 ± 0.8 µg/kg). Furthermore, significant variation (P < 0.01) was observed with maize samples from Osijek having higher OTA mean content than those from Hrvatsko Zagorje and Istria. This clearly demonstrates a significant regional variation in maize contamination by OTA in Croatia which probably reflects variability of climatic conditions in our country. 

In a more recent study aimed to determine the degree of co-contamination of OTA, FB_1_ and zearalenone (ZEA), Domijan *et al.* [22] collected 49 maize samples from all counties across Croatia. In this case, mean OTA level in positive samples (39%) was low (1.5 µg/kg) and thus, OTA contamination of maize in our country is not the significant source of human exposure to OTA. However, further analyzes revealed that all samples (100%) analyzed were contaminated with FB_1_ (mean content 459.8 µg/kg) with 84% of these samples having ZEA at mean level of 3.8 µg/kg. Therefore, only 8% of samples were contaminated with one mycotoxin (FB1), 55% with two and 37% with three mycotoxins. The mycological analysis revealed that high percentage of maize samples collected in our country is contaminated with *Fusarium* spp. and *Penicillium* spp.; while *Aspergillus* spp. is not that frequent [23]. *Aspergillus* spp. were found in five out of 15 samples.

### 2.3. Beans

Beans (*Phaseolus vulgaris* L.) are a staple food consumed in all parts of Croatia. They are usually desiccated and preserved to be consumed during winter. The first reports on OTA contamination of beans as part of the studies in the EN region of Croatia revealed that beans might be the source of the OTA exposure [12,24]. In more recent study samples of dry beans (N = 45) were collected shortly after the harvest from 13 bean-growing counties of Croatia [25]. Data obtained in this study, showed that the mean OTA concentration in all bean samples was quite low (0.41 ± 0.21 µg/kg), ranging between 0.25–0.92 µg/kg. In these samples, OTA was found in 38% of samples, contaminated either with *Penicillium* and *Aspergillus* spp. Furthermore, it was found that samples from the southern Croatia (the Mediterranean part) were less contaminated with these moulds and found to be OTA-free. These bean samples were not stored for longer periods and thus, the presence of OTA in these samples may be associated with field contamination. As found in these samples, the highest incidence rate was found in samples from the north (5/7 samples), followed by those east of Croatia (six out of 13) having a continental climate as well as higher summer and autumn humidity conditions relative to those from other counties. Except in the samples from southern Croatia that were OTA free, in the other regions the mean OTA concentration was from 0.3 to 0.54 µg/kg.

### 2.4. Grapes and Wine

OTA frequently contaminates wine [26] contributing significantly to human exposure to this mycotoxin [27]. It was estimated that 15% of daily intake of OTA is due to wine consumption [5]. 

The first reported study on commercial wine samples from Croatia was provided by Domijan *et al.* [28]. Analysis was performed using HPLC with fluorescence detection. In this study it was confirmed that red wine contains higher OTA concentrations (mean ± SD; 20 ± 11 ng/L) and showed a higher frequency (7/7), compared with white wine, which had lower incidence rate (4/7) and an OTA mean level of 10 ± 9 ng/L (detection limit of 10 ng/L). Despite the low number of analyzed samples a geographical distribution of OTA in wine was seen. Accordingly, all samples collected in south Croatia (the Mediterranean part) contained OTA, unlike in the continental (north) part of Croatia where some samples did not contain OTA. Although a high proportion of wine samples contained OTA, concentrations in these samples were lower than those from neighbouring countries (Austria and Italy)[5,26]. In another study red must samples as well as wine produced from these musts were analyzed [29]. As expected, red must samples contained OTA in higher concentrations than the matching wines produced from them. *Aspergillus carbonarius*, the most common OTA producer in grapes was not detected and it is therefore, likely that in Croatia, OTA on grapes is produced by some other fungal spp.

**Table 1:** Ochratoxin A contamination of various commodities from Croatia.

**Commoditiy**	**Sampling year**	**Sampling region**	**Sample no. positive**	**Mean SD**	**Range**	**References**
			**Ochratoxin A (μg/kg) ^a^**			
Maize	1972–1976	EN	45/542	- ^1^	- ^1^	[19]
	1996	EN	4/45	0.7 ^2,3^	0.36–1.1	[21]
		non-EN	6/60	62.7 ^2,3^	0.36–223.6	[21]
	1997	EN Slavonski Brod	25/50	73.4 ^2,3^	0.29–613.7	[21]
		non-EN	11/56	20.2 ^2,3^	0.26–216.6	[21]
	1999	EN Slavonski Brod	9/11	20.0 **±** 14.8	0.02–40.0	[20]
		non-EN Osijek	- ^1^	0.8 **±** 1.3	0.02–4.0	[20]
		non-EN Istria	- ^1^	0.4 **±** 0.8	0.02–1.6	[20]
		non-EN Hrvatsko Zagorje	- ^1^	0.4 **±** 0.4	0.02–2.0	[20]
	2002	non-EN	19/49	1.47 **±** 0.38 ^3^	- ^1^	[22,23]
Wheat	1999	EN Slavonski Brod	33/33	38.7 ± 27.1	2.0–160.0	[20]
		non-EN Osijek	24/27 ^b^	8.7 ± 8.2	0.02–32.0	[20]
		non-EN Istria	- ^1^	1.3 ± 2.6	0.02–6.4	[20]
		non-EN Hrvatsko Zagorje	- ^1^	2.1 ± 1.5	0.02–4.8	[20]
	1972–1976	EN	11/130	- ^1^	5–140	[19]
Beans	2001	non-EN	17/45	0.41 ± 0.21 ^3^	0.25–0.92	[25]
Red wine	2002–2003	non-EN	7/7	22 ± 11	12–47	[28]
White wine	2002–2003	non-EN	4/7	10 ± 9	15–22	[28]
Red wine	2007	non-EN	6/6	- ^1^	19–50	[29]
White wine	2007	non-EN	8/10	- ^1^	2–21	[29]

^1^ data not available; ^2^ SD not available; ^3^ positive samples. ^a^ The concentrations of OTA in wine is measured in ng/mL; ^b^ No. of positive samples (24/27) is for those collected in 1999 in non EN regions. EN: Endemic nephropathy; non-EN: Region with no endemic nephropathy.

## 3. Conclusions

Studies on OTA contamination of commodities produced in Croatia have revealed the presence of this mycotoxin. In recent studies using sensitive methods even very low OTA concentrations could be detected resulting in increased number of positive samples. Regardless of the limited number of samples studied herein this review provides an up-date confirmation that OTA contaminates various commodities from Croatia. The highest OTA contamination was found in maize samples, usually not intended for human consumption. Apart EN region, the levels of OTA in wheat, beans and wine are low, suggesting non-significant human exposure to this mycotoxin. This is in accordance with our previous study where the human exposure to OTA was estimated by OTA quantification in human blood collected out of the EN region in Croatia [30]. However, if humans are exposed even to such low levels of OTA together with other mycotoxins, this may result in synergistic or additive effects in inducing genotoxicity and/or carcinogenicity and thus severely affect human health, as revealed by results on experimental animals. In the available literature such studies on human exposure to more than one mycotoxin are rare and should be encouraged. 
